# 

**Published:** 1871-03

**Authors:** 


### CONTENTS:

ORIGINAL COMMUNICATIONS:

ARTICLE I. Some remarks upon Cases of Obstruction of the Bowels, in which Surgical Means were used. By A. Reeves Jackson, M.D. 129

ART. II. Case of Preternatural Labor, Complicated with Convulsions and an Hour-Glass Contraction of the Uterus. By D. W. Flick, M.D. 135

ART. III. On a few Instruments for the Practical Treatment of Uterine Disease. By Philip Adolphus, M.D. .......... 138

ART. IV. Our Officinal Cathartic Pills. By E. Ingals, M.D .......... 147

ART. V. Cook County Hospital. Reported by Curtis T. Fenn, M.D.. 171

RUSH MEDICAL COLLEGE — ALUMNI MEETING AND COMMENCEMENT EXERCISES .......... 149

FOREIGN CORRESPONDENCE .......... 176

EDITOR'S BOOK TABLE .......... 183

EDITORIAL .......... 185

NEWS AND GOSSIP .......... 188

LOOT .......... 192

